# Programmed Molecular Assembly of Abrupt Crystalline Organic/Organic Heterointerfaces Yielding Metal‐Organic Framework Diodes with Large On‐Off Ratios

**DOI:** 10.1002/advs.202001884

**Published:** 2021-01-21

**Authors:** Abhinav Chandresh, Xiaojing Liu, Christof Wöll, Lars Heinke

**Affiliations:** ^1^ Karlsruhe Institute of Technology (KIT) Institute of Functional Interfaces (IFG) Hermann‐von‐Helmholtz‐Platz 1 Eggenstein‐Leopoldshafen 76344 Germany

**Keywords:** crystalline heterointerfaces, metal‐organic frameworks, organic‐organic heterojunctions, p–n junctions, thin films

## Abstract

Structurally well‐defined, crystalline organic/organic heterojunctions between C_60_‐ and anthracene‐based semiconductors are realized via layer‐by‐layer deposition of metal‐organic framework, MOF, thin films. As demonstrated by X‐ray diffraction, perfect epitaxy is achieved by adjusting the lattice constants of the two different MOFs. Deposition of top electrodes allows to fabricate p–n as well as n–p devices. Measurements of the electrical properties reveal the presence of high‐performance diodes, with a current on/off ratio of up to 6 orders of magnitude and an ideality factor close to unity. The crystalline nature of the abrupt organic/organic heterojunction provides the basis for a rational, simulation‐based optimization and tailoring of such organic semiconductor interfaces.

## Graphical abstract

Crystalline abrupt organic/organic heterojunctions are presented. This is realized by incorporating semiconducting molecules, here anthracene and C_60_, in metal‐organic framework thin films fabricated in a layer‐by‐layer fashion forming bilayer films. The p–n‐junctions show strong diode performance with a current rectification of up to six orders of magnitude.

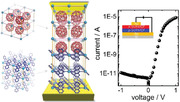

Semiconducting organic materials feature striking advantages over inorganic materials like silicon or GaAs in certain fields of application, e.g. in the context of printed electronics, light‐emissive devices, or photovoltaics, as well as in the context of stretchable electronics and photosensors.^[^ ^1^, ^2^, ^3^, ^4^, ^5^ ^]^ Popular organic semiconductor (OSC) materials are based on fullerenes or polycyclic aromatic hydrocarbon like anthracene, pentacene and rubrene. Fullerenes, like C_60_, are excellent n‐conductors.^[^ ^6^ ^]^ C_60_ is used as active component in numerous organic n‐type materials for various organic electronic applications like organic solar cells and molecular electronics.^[^ ^6^, ^7^, ^8^, ^9^ ^]^ Among p‐conducting OSC materials, anthracene has a very low electron affinity.^[^ ^10^ ^]^ Anthracene‐based materials find numerous application as organic p‐type semiconductor.^[^ ^10^, ^11^, ^12^ ^]^

For the performance of semiconducting devices, the interface between two materials is pivotal. This was coined in the phrase “the interface is the device” for inorganic semiconductors,^[^ ^13^, ^14^ ^]^ but it is also true for organic materials.^[^ ^15^ ^]^ In analogy to inorganic semiconductors, the fabrication of all‐organic p–n junctions from such n‐ and p‐conducting OSC materials should be straightforward. Such devices have been theoretically predicted,^[^ ^16^ ^]^ and were demonstrated experimentally for single molecules^[^ ^17^ ^]^ and polymers.^[^ ^18^ ^]^ The fabrication of crystalline OSC p–n heterobilayers with well‐defined interfaces has not yet been possible, because the available fabrication methods, e.g. molecular beam deposition or solution‐based approaches (like spin coating), do not result in well‐defined, stable interfaces with long‐range crystalline order.^[^ ^2^, ^4^ ^]^ As a result, even very simple devices like an organic diode with a crystalline interface have not been presented to date.

With regard to positioning organic compounds in a perfect, crystalline lattice exhibiting long‐range order, metal‐organic frameworks, MOFs, offer unique opportunities. MOFs are a class of crystalline, nanoporous hybrid materials, formed by connecting di‐ or higher‐topic organic linkers via metal nodes.^[^ ^19^ ^]^ Since the modification of organic molecules to function as MOF linkers is straightforward, these crystalline frameworks have attracted particular attention in the field of optical^[^ ^20^ ^]^ and electronic applications.^[^ ^21^, ^22^ ^]^ The electronic conductivity of MOFs can be tuned over many orders of magnitude by loading with suitable molecules, such as tetracyanoquinodimethane (TCNQ).^[^ ^23^ ^]^ By a diffuse doping gradient of TCNQ in the film, a diode‐like current rectification was demonstrated.^[^ ^24^ ^]^ With respect to electronic applications, field‐effect transistors,^[^ ^25^ ^]^ solar cells^[^ ^26^ ^]^ and light‐emitting diodes^[^ ^27^, ^28^ ^]^ with MOFs as active layer were realized. The efficiency of the presented devices is far from ideal, also due to the poorly‐defined interface between the layers. Further advanced semiconducting devices based on MOFs, like lasers, were not yet presented due to the lack of defined heterointerfaces in multi‐layered functional MOF films.

The realization of an all‐MOF abrupt heterointerface is most easily accomplished by using the MOF‐on‐MOF approach.^[^ ^29^ ^]^ While the realization of well‐defined heterointerfaces is difficult with the conventional solvothermal methods used to fabricate the common bulk form of MOF materials, layer‐by‐layer (lbl) methods^[^ ^30^ ^]^ allow for a straightforward realization of MOF‐on‐MOF architectures. In the past, such lbl methods have been successfully used to fabricate homogeneous films of surface‐mounted MOF thin films with defined thicknesses, referred to as SURMOFs.^[^ ^30^ ^]^ Modification of the lbl approach to realize hetero‐SURMOFs is straightforward.^[^ ^31^, ^32^, ^33^ ^]^ While the domain sizes in the SURMOF film are typically in the µm range, cm‐sized domains can be prepared by using appropriate substrates.^[^ ^32^ ^]^ In addition, the lbl process allows to control defect densities^[^ ^34^ ^]^ – a key requirement in the field of organic semiconductors.^[^ ^35^ ^]^

Here, we present MOF diodes containing p–n and n–p junctions between anthracene‐ and fullerene‐based SURMOFs. The lattice parameters of the two molecular frameworks were adjusted to yield true epitaxy, thus obtaining strain‐free heterojunctions of high structural quality. Determination of the electrical properties of the p–n and n–p junction thin films reveals clear diode‐like behavior with current on/off ratios of up to six orders of magnitude. The ideality factor, an important figure of merit for semiconductor junctions, was close to unity, indicating a high structural quality of the interface with a low density of charge traps.

Crystalline anthracene‐ and fullerene‐containing SURMOFs, **Figure** **1**a,b, were grown on functionalized gold substrates using the lbl method. By X‐ray diffraction (XRD), Figure 1e,f, it was found that the SURMOFs exhibit a pillared‐layer structure.^[^ ^29^ ^]^ In the following, Cu_2_(adc)_2_(dabco) and C_60_@Cu_2_(bdc)_2_(dabco) are referred to as p‐SURMOF and n‐SURMOF, respectively. adc denotes 9,10‐anthracene dicarboxylate, bdc is 1,4‐benzene dicarboxylate and dabco is 1,4‐diazabicyclo[2.2.2]octane. The comparison of the C_60_‐containing n‐SURMOF with the C_60_‐free reference SURMOF, Figure S2 (Supporting Information), shows a clear shift of the intensity ratio of the diffraction peaks. This change of the XRD‐form‐factor clearly indicates that the electron density differs, most likely due to the embedment of C_60_. An estimation, see Figure S2 (Supporting Information), indicates that each SURMOF pore is filled with one C_60_. The C_60_ loading is also confirmed by UV–vis spectroscopy, Figures S4, S6, and S7 (Supporting Information), as well as by Raman spectroscopy, Figure S5 (Supporting Information). The lattice parameters of both SURMOF structures are identical, with distances of 1.06 nm, 1.06 nm, and 0.95 nm between the (100), (010) and (001) lattice planes, respectively.^[^ ^29^, ^36^ ^]^ The diffractograms measured in out‐of‐plane geometry (Figure 1e) also show that all SURMOF films are grown in [001] direction perpendicular to the substrate surface. This finding is verified by the diffractograms recorded in the in‐plane geometry (Figure 1f) which show all diffraction peaks perpendicular to (001). In addition, the identical positions of the reflexes in the in‐plane diffractograms show that the lattice parameters parallel to the interface are equal and the MOF structures match on top of each other without strain.

**Figure 1:**
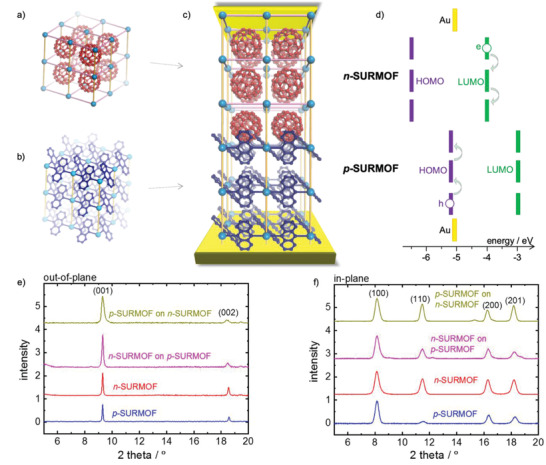
Sketch of fullerene‐containing n‐SURMOF a) and of anthracene‐containing p‐SURMOF b) with 2 × 2 × 2 unit cells. c) The bilayer p–n‐SURMOF with the crystalline heterointerface and the top and bottom gold electrodes. d) The energy levels of the p–n‐junction SURMOF with the HOMO and LUMO levels of the p‐ and n‐SURMOF. The out‐of‐plane e) and the in‐plane f) X‐ray diffractograms of the single‐ and bi‐layered SURMOFs. The sample names and the diffraction peaks are labelled. The comparisons of the recorded data with the calculated diffractograms as well as the stick‐and‐ball structure of the SURMOFs are shown in Figure S1 in the Supporting Information.

The rather homogenous film morphology can be seen on the scanning electron microscopy (SEM) images in Figure S3 in the Supporting Information. The film thickness is estimated to be about 100 nm. Moreover, the film shows no pinholes, which would cause shortcuts when measuring the conduction in top‐down geometry.

The conduction of the SURMOF films were measured in a top‐down (also referred to as cross‐plane) geometry in a pure nitrogen atmosphere, avoiding contributions from volatile guest molecules like water. The bottom electrode is the gold film of the substrates and the top electrode is a thin gold‐disc, gently deposited on the SURMOF. In this way, the bottom and top electrode are made of the same inert material where oxide formation can be excluded. Based on the crystalline orientation, the current along the [001] direction is measured. The current–voltage curves of the single SURMOF films are shown in Figures S8 and S12 in the Supporting Information. The current–voltage curves of a Cu_2_(bdc)_2_(dabco) reference sample, which has the same pillared‐layer structure but does not contain anthracene or fullerene, shows current values which are three orders of magnitude smaller than of the anthracene‐ or fullerene‐containing samples. This indicates that the conduction paths along anthracene and fullerene, respectively, dominate the conduction in the p‐ and n‐SURMOFs. Based on the HOMO (highest‐occupied molecular orbital) level of adc of about ‐5.25 eV^[^ ^10^ ^]^ and the work function of the gold electrodes of approximately 5.1 eV, we conclude the p‐SURMOF is electron‐hole conducting, in line with common anthracene‐based materials.^[^ ^10^, ^11^, ^12^ ^]^ The LUMO (lowest unoccupied molecular orbital) level of C_60_ is approximately ‐4 eV,^[^ ^37^ ^]^ hence, the n‐SURMOFs is electron conducting, as usually found for C_60_‐based materials.^[^ ^6^, ^7^, ^8^, ^9^ ^]^ The energy levels are sketched in Figure 1d. Based on the fact that anthracene and fullerene are the active moieties, we conclude that the electronic conduction happens via charge hopping, as common for organic semiconductor at room temperature.^[^ ^38^, ^39^ ^]^ The charge hopping between the frontier orbitals of the organic molecules can be described by Marcus theory.^[^ ^40^, ^41^ ^]^

For designing and fabricating p–n‐junctions, Figure 1c, the anthracene‐ and fullerene‐containing SURMOFs are grown on top of each other. The X‐ray diffractograms, see Figure 1e,f, show that the multilayered films are grown in (001) orientation, like the pure films. Noteworthy, the increase of XRD peak intensity points on the increase of the SURMOF material when growing the p‐SURMOF on the n‐SURMOF and vice versa. The fact that both films have the same lattice parameters allows true epitaxial growth of both films on each other, without distortion of the lattice. Due to size reasons, C_60_ cannot diffuse into the p‐SURMOF, hindering n‐doping of the p‐SURMOF, resulting in an abrupt heterojunction. It should be noted that the SURMOF‐p–n‐heterojunction is based on coordinative bindings which is significantly stronger than weak van‐der‐Waals interaction which is typically the basis for organic/organic p–n‐junctions of small molecules.

The current–voltage curves of the bilayer p–n‐SURMOF are shown in **Figure** **2**a. While the current at 1 V in forward‐bias direction amounts to 8.1 µA, it is only 11.2 pA in reverse‐bias direction. Thus, the p–n‐bilayer‐SURMOF shows a diode‐like behavior with a rectification ratio of approximately 6 orders of magnitude. According to the Schottky model, the current across a p–n‐junction can be described by

(1) $$I=I_{0}\;\left(\exp\left(\frac{\mathrm{qU}}{n_{\mathrm{id}}k_{B}T}\right)-1\right)$$

where *q* is the charge (+ or – the elemental charge *e*), *k*_B_ is the Boltzmann constant and *T* the temperature. *n*_id_ denotes the ideality factor. Here, an ideality factor of 1.2 was obtained for the p–n‐heterojunction (see Figure 2a). An ideality factor close to unity indicates that the p–n interface is of high structural quality with a low density of charge traps.^[^ ^42^ ^]^

**Figure 2:**
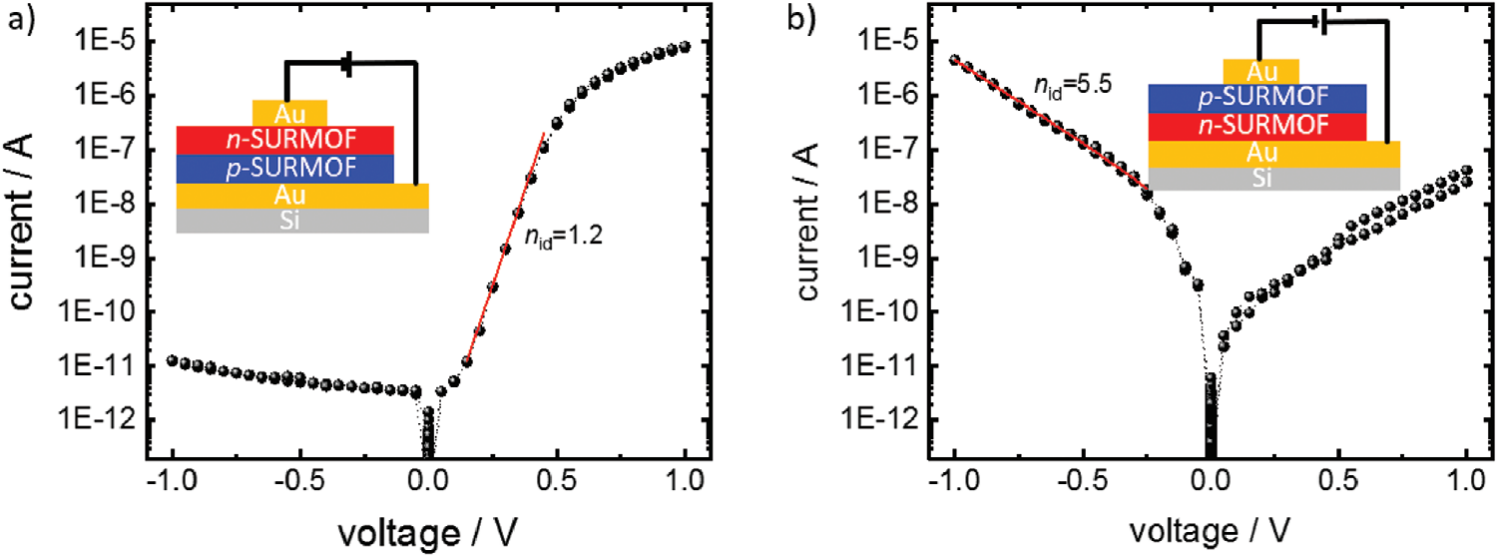
Current–voltage curves of the p–n‐SURMOF a) and of the n–p‐SURMOF b). The setup is sketched. The curves are measured in 2 consecutive voltage cycles, resulting in virtually identical current values. The thin red lines indicate the linear regions in the log‐plots with the corresponding ideality factors. Current‐curves for voltages up to 3 V are shown in Figure S9 in the Supporting Information.

When changing the deposition sequence of the two different SURMOFs, thus realizing n–p instead of p–n junctions, there is again a pronounced difference between forward and reverse direction, 4.6 µA versus 25.5 nA, yielding an on‐off ratio of about 200. We believe that the lower performance of the n–p junctions results from the asymmetry of the heterointerface. We propose that the number of defects is higher for growing the p‐SURMOF on top of the n‐SURMOF than vice versa. This hypothesis is supported by the fact that the roughnesses of individual n‐SURMOFs are larger than that of p‐SURMOFs, see Figure S3 in the Supporting Information. In line with this hypothesis, the ideality factor for the n–p device was found to be much larger than 1, here 5.5. The device performance was reproduced with different samples, Figures S10 and S11 (Supporting Information), where similar current rectifications were observed.

The current densities of the devices in forward direction at 1V are in the range of 1 mA cm^−2^, which is somewhat smaller that the current density in other organic p–n‐junctions.^[^ ^43^, ^44^ ^]^ Since the current density is estimated under the assumption that the Au‐preform top electrode builds a solid, homogenous contact to the SURMOF (neglecting any SURMOF surface roughness), it might be argued that the actual current density is slightly higher and larger current density will be obtained with different electrodes. Based on the current–voltage curves, we estimate a small knee voltage of about 0.1 V.

In control experiments, for devices containing only single component n‐ or p‐conducting SURMOFs, simple ohmic behavior without current rectification was observed, see Figure S8 in the Supporting Information.

In conclusion, crystalline molecular framework bilayers made of two different organic semiconductors, anthracene and fullerene, with a well‐defined abrupt organic/organic heterojunction were prepared using a layer‐by‐layer approach. Combining n‐conducting fullerene‐based and p‐conducting anthracene‐based SURMOFs yields p–n‐heterojunctions with crystalline interfaces and low numbers of defects. By adding top electrodes, functioning devices were obtained, showing a diode on/off ratio of up to six orders of magnitude.

Taking advantage of the huge chemical space spanned by MOFs and SURMOFs containing organic semiconducting molecules, the present approach can be used to prepare an enormous amount of structurally well‐defined heterojunctions. Since the structures are well known, theoretical methods can then be applied to select promising materials by computational screening.^[^ ^45^ ^]^ In particular, we expect that such approaches will contribute to the rational design of all‐MOF‐based devices with controlled interfaces, like light emitting diodes, semiconductor lasers and photovoltaic cells. Further improvement of the device performance is also expected by different electrode materials and more sophisticated methods for their depositions.^[^ ^46^, ^47^ ^]^

## Experimental Section

The semiconducting films were grown on gold substrates, that were gold thin films on silicon wafers purchased from PVD‐*Beschichtungen*, Silz, Germany. The gold substrates were functionalized with a 11‐Mercapto‐1‐undecanol (Sigma Aldrich) self‐assembled monolayer to support and direct the SURMOF growth. Thin MOF films were synthesized in layer by layer (lbl) fashion as previously discussed in detail.^[^ ^30^, ^48^ ^]^ Briefly, the samples were alternatively immersed in the ethanolic metal node solution (here: 1 × 10^−3^ m copper acetate, Alfa Aesar) and in the ethanolic linker solutions (here: 0.1 × 10^−3^ m dabco, 1,4‐diazabicyclo[2.2.2]octane, Merck KGaA, and 0.1 × 10^−3^ m bdc, benzene‐1,4‐dicarboxylic acid, Sigma Aldrich, or 0.1 × 10^−3^ m adc, 9,10‐anthracene dicarboxylate, Sigma Aldrich) for 5 min and 15 min, respectively. In between, the samples were cleaned with pure ethanol for 1 min. For the synthesis of C_60_@Cu_2_(bdc)_2_(dabco), the SURMOF samples were immersed in the C_60_ solution (0.5 mg/ml in toluene, Sigma Aldrich) after each linker step, resulting in a step‐by‐step SURMOF growth with an simultaneous C_60_ embedment.^[^ ^49^ ^]^ A dipping robot was used for the SURMOF fabrication.^[^ ^50^ ^]^ Each SURMOF was prepared in 50 synthesis cycles. The p–n SURMOFs were prepared in 50 cycles of Cu_2_(adc)_2_(dabco) (p‐SURMOF) followed by 50 cycles of C_60_@Cu_2_(bdc)_2_(dabco) (n‐SURMOF), and vice versa for n–p.

The X‐ray diffractograms in out‐of‐plane geometry were measured with a Bruker D8‐Advance diffractometer equipped with a position‐sensitive detector in *θ*–*θ* geometry. The in‐plane X‐ray diffractogram measurements were carried out with a Bruker D8 Discover. In both cases, Cu‐anodes with a wavelength of *λ*  =  0.154 nm were used.

Raman spectroscopy was performed with a Bruker Senterra Raman microscope, equipped with an 50xOlympus MPLAN objective and a 532nm‐laser, operated at 200µW output power. For data acquisition and spectra analysis Bruker OPUS software 7.8 was used. The spot size for C_60_ powder and C_60_ in SURMOF was 100 µm^2^. For the Raman spectroscopy of the SURMOF, the spectra were recorded at 5 different positions on the sample and their intensities were added.

UV–vis transmission spectra were recorded with a Cary5000 spectrometer from Agilent.

Electrical conduction characterization was carried out using a Keithley 2635B source meter in combination with a CascadeMicrotech probe station and a home‐built cell allowing the conduction measurement of the samples in pure N_2_ atmosphere. The bottom contact to the film was made by electrically contacting the gold substrate. The top contact was made by depositing a gold preform, that was a thin gold disc of 1.26 mm diameter and 10 µm thickness. The gold preforms were purchased from AIM solder. The contacts between the gold bottom and top electrodes with the needles of the probe station (connecting the source meter) were improved by using indium patches. The maximum area of the top electrode was 0.012 cm^2^, assuming a solid contact between the SURMOF and the entire area of the top electrode.

All measurements were done at room temperature.

Scanning electron microscopy (SEM) images were recorded with a TESCAN VEGA3 tungsten‐heated‐filament SEM and a Zeiss Leo 1530 microscope. To avoid charging effects, the samples were coated with a thin (≈5 nm) Au/Pd film. The samples have been imaged under high vacuum conditions and using an acceleration voltages of 5 and 10 kV.

##### Statistical Analysis

All shown data are representative for the samples. The UV–vis spectra and current–voltage curves are shown in its pristine form, that means without any data manipulation. The Raman spectra as well as the X‐ray diffractogram are shown after subtracting the baselines of the spectra for a better visibility. At the X‐ray diffractogram, the correct calibration and sample position is confirmed by measuring the Au(111) diffraction peak. The current voltage curves are shown without manipulation in Figure 2 and Figures S10 and S11 (Supporting Information). In addition, several samples were recorded which showed short‐cuts, which were not further evaluated. For the SEM images, many SEM images were recorded and Figure S3 in the Supporting Information gives representative images of the samples.

## Conflict of Interest

The authors declare no conflict of interest.

## Supporting information

Supporting Information
