# Supplementary material for: Programmed Molecular Assembly of Abrupt Crystalline Organic/Organic Heterointerfaces Yielding Metal‐Organic Framework Diodes with Large On‐Off Ratios
Source: Adv Sci (Weinh). 2021 Jan 21;8(7):2001884. doi: 10.1002/advs.202001884 (PMC8024988; doi:10.1002/advs.202001884)
Supplement: Supplementary file 1 — Supporting Information [file ADVS-8-2001884-s001.pdf]

WILEY-VCH

© 2020 Wiley-VCH GmbH

ADVANCED SCIENCE
Open Access

# Supporting Information

for *Adv. Sci.*, DOI: 10.1002/advs.202001884

# Programmed Molecular Assembly of Abrupt Crystalline Organic/Organic Heterointerfaces Yielding Metal-Organic Framework Diodes with Large On-Off Ratios

*Abhinav Chandresh, Xiaojing Liu, Christof Wöll, Lars Heinke**

# Supporting Information

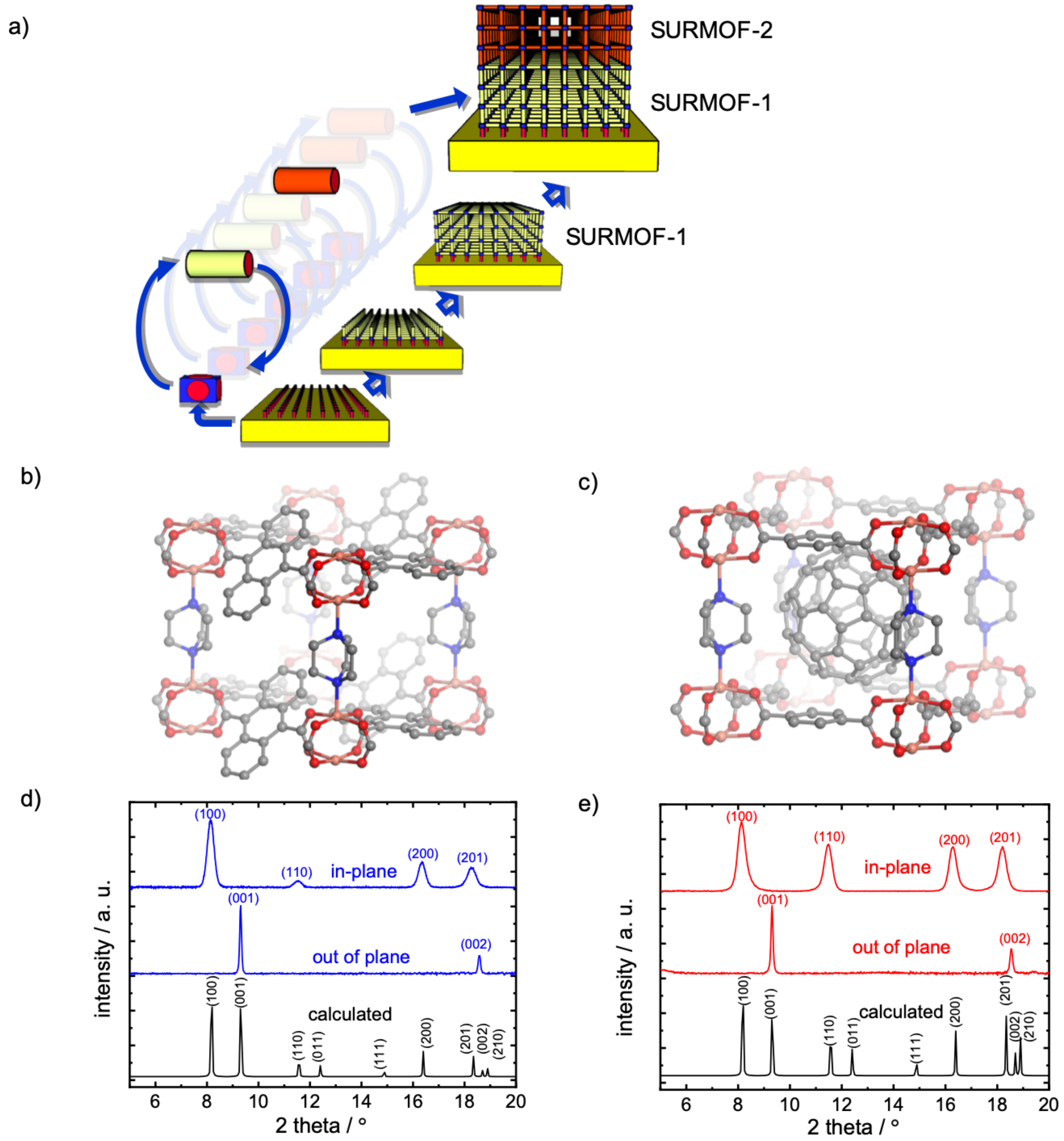


Figure S1. a) Sketch of the layer-by-layer SURMOF synthesis. The exchange of the MOF components during the synthesis results in bilayer-SURMOFs. The molecular stick-and-ball model of the *p*-SURMOF, that is $Cu_2(adc)_2(dabco)$ (b), and of the *n*-SURMOF, that is $C_{60}@Cu_2(bdc)_2(dabco)$ (c). Carbon is plotted in grey, oxygen red, copper orange, nitrogen blue and hydrogen is not shown. The recorded out-of-plane and in-plane X-ray diffractograms as well as the calculated data with the diffraction peaks for all directions of the *p*-SURMOF (d) and *n*-SURMOF (e).

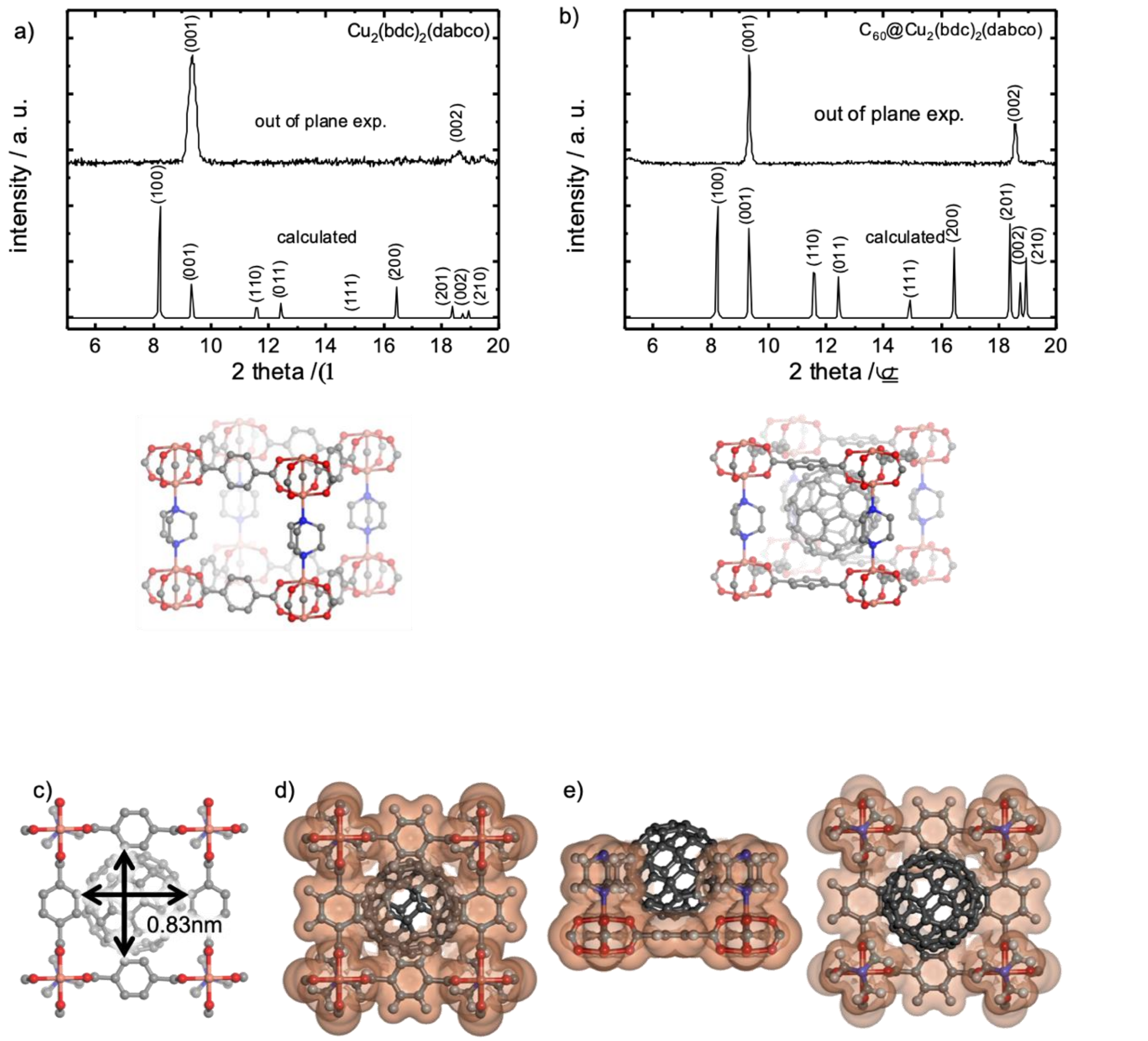


Figure S2. Out-of-plane X-ray diffractograms (XRDs) of $Cu_2(bdc)_2(dabco)$ (a) and of $C_{60}@Cu_2(bdc)_2(dabco)$, i.e. *n*-SURMOF,(b) compared with the calculated diffractograms. The MOF unit cells used for the calculations of the diffractograms are shown below. For $Cu_2(bdc)_2(dabco)$, the intensity ratio of the (001):(002) peaks is approximately 8.5 measured, compared to 7.0 in the calculated XRD. For $C_{60}@Cu_2(bdc)_2(dabco)$, the intensity ratio of the (001):(002) peaks is 2.73 measured, compared to 2.77 in the calculated XRD. The shift of the XRD-form-factor as well as the very good agreement of the intensity ratios of the *n*-SURMOF indicate that the fullerenes are incorporated in essential all MOF unit cells.
c) Sketch of $C_{60}@Cu_2(bdc)_2(dabco)$ with the distance between the carbons limiting the pore entrance indicated. The hydrogen atoms as well as the van-der-Waals radii are not indicated.
d) Sketch of the MOF structure with the van-der-Waals-surface. View along the [001] direction (top view). The pore windows are significantly smaller than $C_{60}$, which has a van-der-Waals-diameter of about 1 nm. Therefore, the passage of $C_{60}$ through the MOF pore windows is sterically hindered.
e) Sketch of the partial MOF structure (i.e. the focus is on the MOF pore) with the van-der-Waals-surface. The structure is shown without the $Cu_2(bdc)_2$-layer closing the MOF pore. The structures are shown for views along [100] (side view) and [001] (top view). The pore body is large enough for the embedment of $C_{60}$.

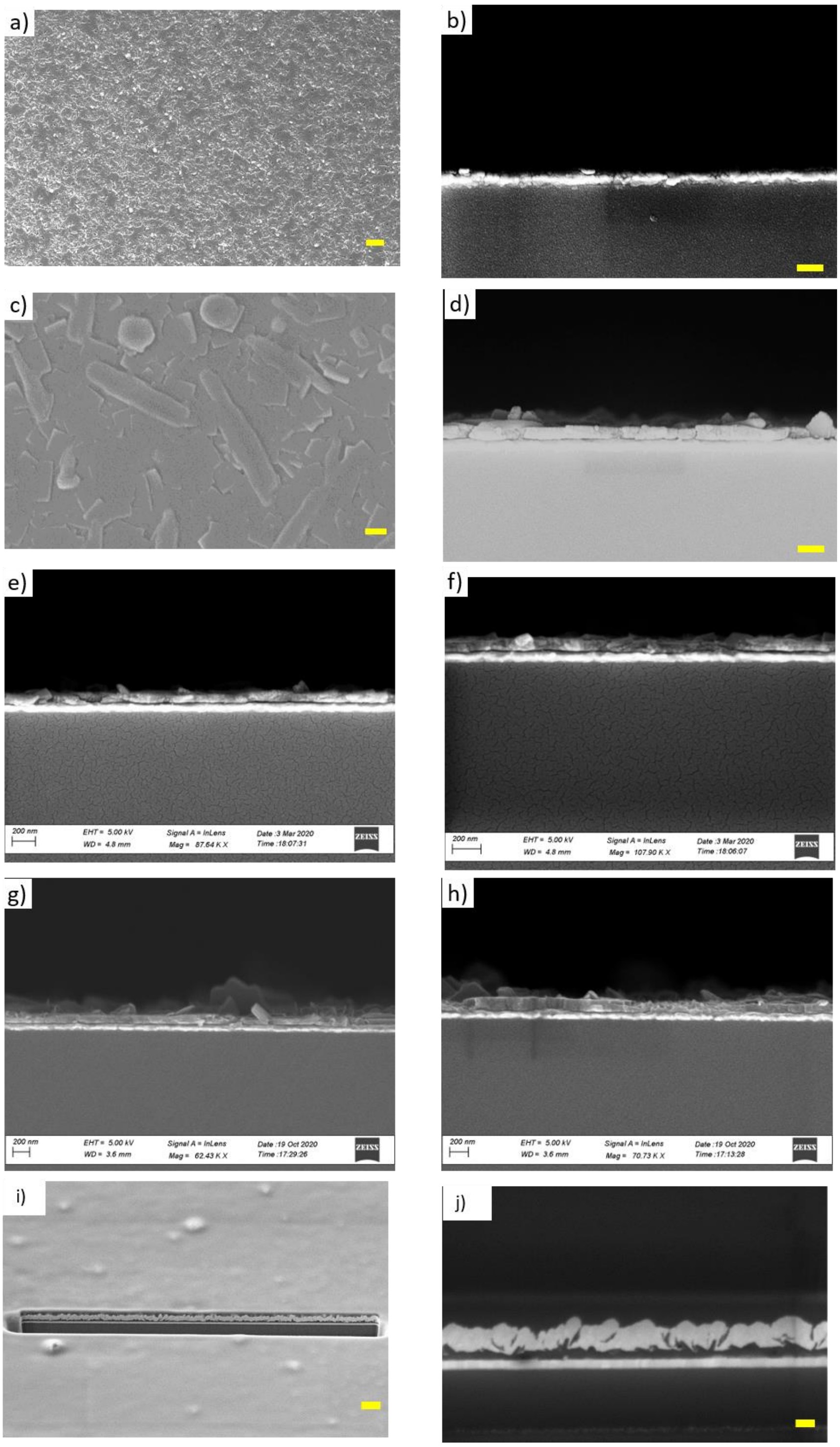


Figure S3. Scanning electron microscopy (SEM) images of the top-view and cross section of the *p*-SURMOF (a and b) and of the *n*-SURMOF (c and d). The cross sections of the *n*-*p*-SURMOF (e and f) and *p*-*n*-SURMOF (g and h). The scale bar, shown in yellow in panels a-d and black in e-f, is 200 nm. The cross-section images (b, c and e-h) were recorded from broken samples. The SURMOF films on the Au@Si-wafer-substrate have thicknesses of approximately 100 nm.  i) and j) SEM images of the *p*-*n*-SURMOF sample cut with a focus ion beam (FIB). Scale bar in (i) is 1 µm, (j) and (c) is 200 nm. Before cutting the sample with the focused $Ga^{+}$-ion beam, a rather thick layer of Au was deposited (bright top layer) protecting the sample. The bilayer structure of the *p*-*n*- or *n*-*p*-SURMOF could not be recognized in the SEM images, presumably due to the very similar MOF structure and composition.

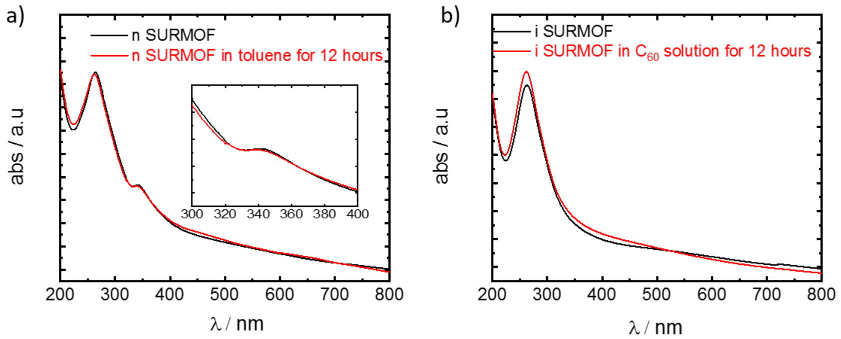


Figure S4: a) UV-vis spectra of $C_{60}$@$Cu_2$(bdc)$_2$(dabco) (*n*-SURMOF) as prepared and upon 12 hours immersion in toluene. b) $Cu_2$(bdc)$_2$(dabco)SURMOF (*i*-SURMOF) as prepared and 12 hours in solution of $C_{60}$ in toluene (1 mg per ml). The UV-vis spectra show that $C_{60}$ cannot be unloaded from the *n*-SURMOF and $C_{60}$ cannot be loaded in a synthesized *i*-SURMOF. Only the layer-by-layer loading[49] during the synthesis results in $C_{60}$@$Cu_2$(bdc)$_2$(dabco). This is also an indication that $C_{60}$ in *n*-SURMOF must be embedded in the SURMOF pores.

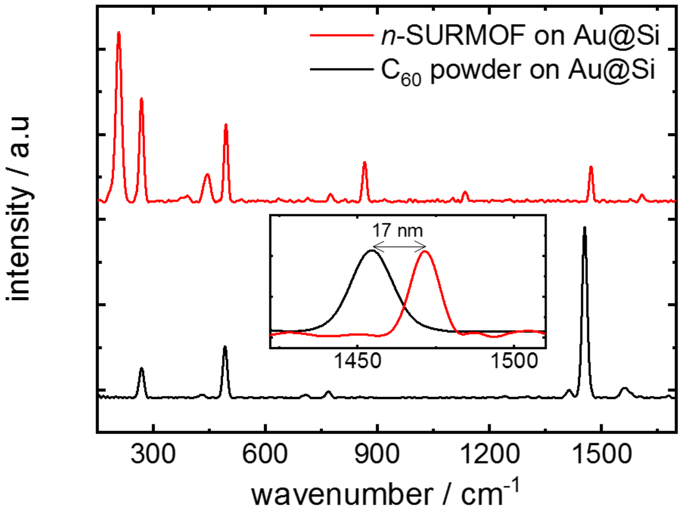


Figure S5: Raman spectra of pure $C_{60}$ powder and $C_{60}$@$Cu_2$(bdc)$_2$(dabco) (*n*-SURMOF). The inset shows a zoom-in of the $A_g(2)$ vibration band.[51-52] For $C_{60}$ in the SURMOF sample compared to pure $C_{60}$ on the same type of substrate (Au@Si-wafer), the band is blue-shifted by 17 $cm^{-1}$.

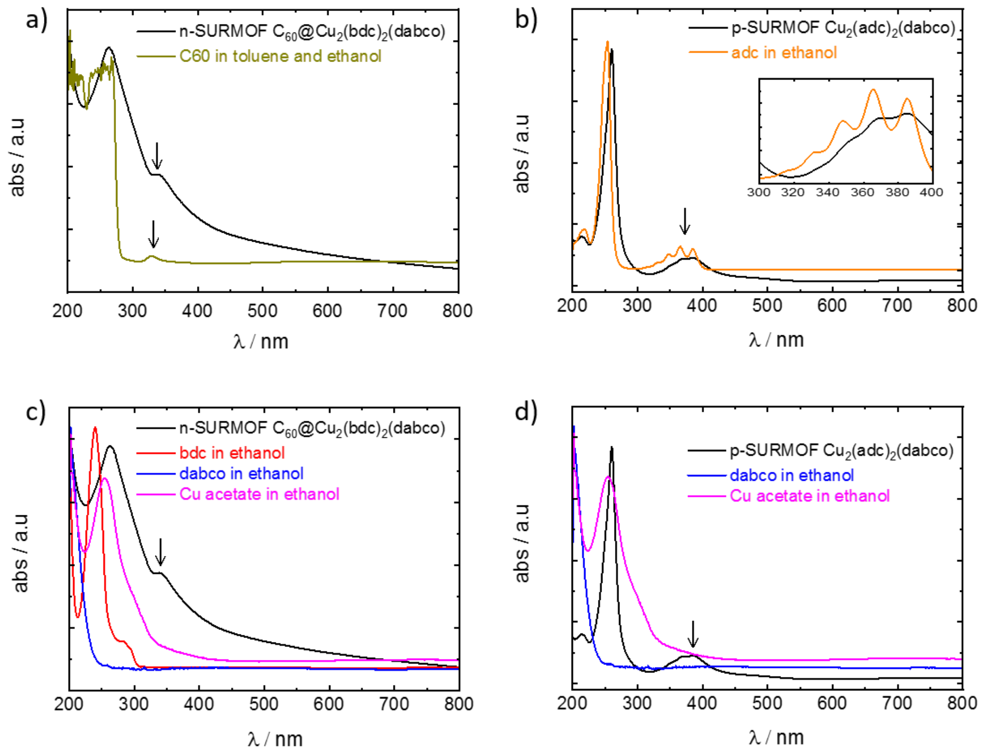


Figure S6: The UV-vis spectra of *n*-SURMOF ($C_{60}$@$Cu_2(bdc)_2$(dabco), a) and *p*-SURMOF ($Cu_2(adc)_2$(dabco), b) with their respective active components ($C_{60}$ in toluene and ethanol mixture for *n*-SURMOF, adc in ethanol for the *p*-SURMOF). c) and d): The comparison of the SURMOF spectra with the inactive components (bdc, dabco and Cu acetate for *n*-SURMOF and dabco and Cu acetate for *p*-SURMOF) in ethanol solution. For $C_{60}$, the band at approximately 340 nm (indicated by arrows) stems from a π-π* transition of the conjugated system.[53] For anthracene, the characteristic bands between 300 nm and 400 nm (indicated by arrows) are also caused by π-π* transitions of the conjugated π-system.[54-55]

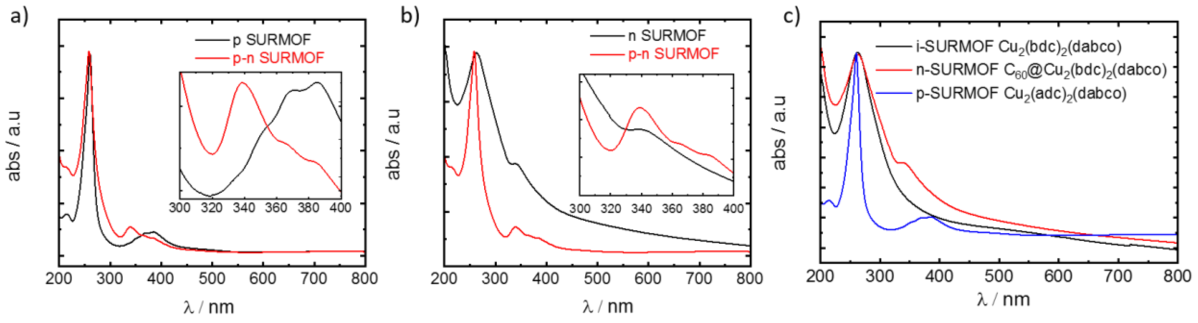


Figure S7: The UV-vis spectra of bilayer *p-n* SURMOF compared with $Cu_2(adc)_2$(dabco) (*p*-SURMOF, a) and $C_{60}$@$Cu_2(bdc)_2$(dabco) (*n*-SURMOF, b). The insets show zoom-ins of the spectra. The active components of *p*- and *n*-SURMOFs are present in the *p-n*-bilayer-SURMOF. c) UV-vis spectra of the *n*- and *p*-SURMOFs compared with the *i*-SURMOF ($Cu_2(bdc)_2$(dabco)) which does not possess any active component. The characteristic bands of $C_{60}$ and of anthracene are visible for all samples containing $C_{60}$ and anthracene, respectively.

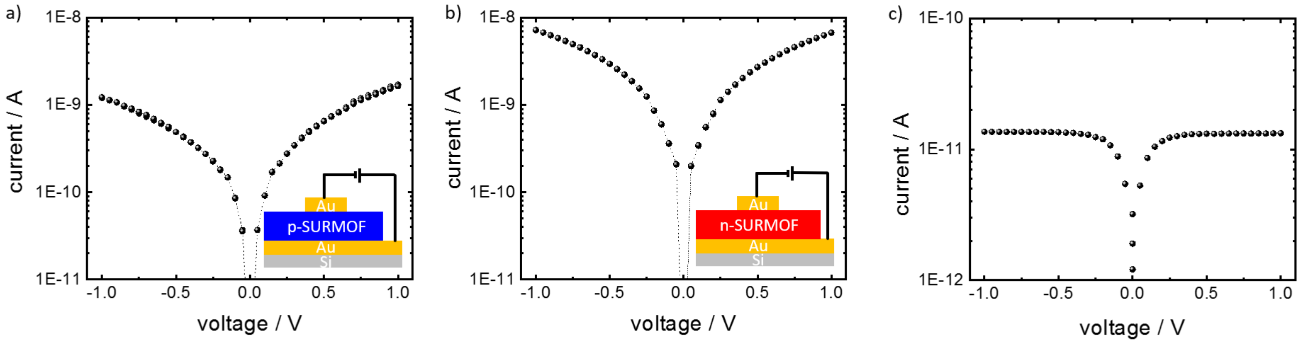


Figure S8: Current voltage curve of pure *p*-SURMOF (a), *n*-SURMOF (b) and $Cu_2(bdc)_2(dabco)$ (c). The curves are measured in 2 consecutive voltage cycles, both resulting in virtually identical current values.

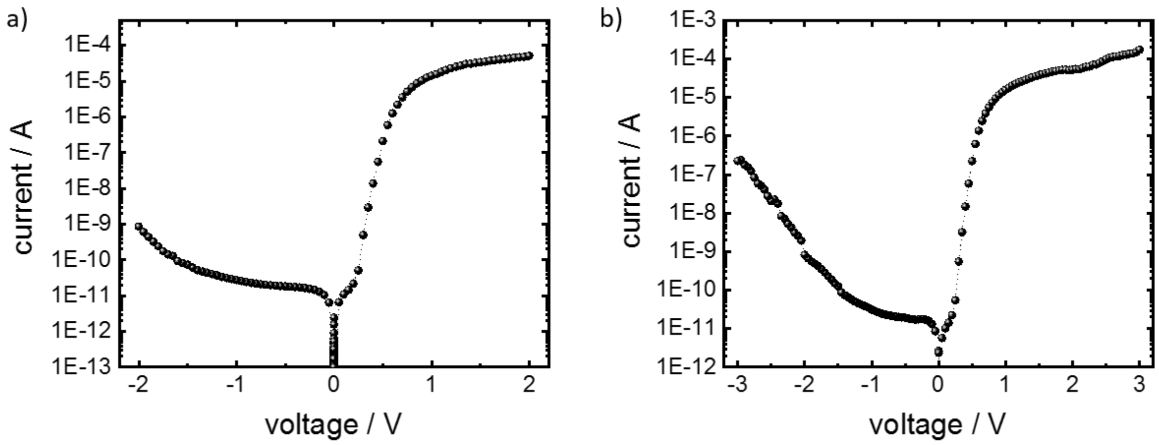


Figure S9: Current-voltage curves of the *p*-*n*-SURMOF for voltage cycles up to 2V (a) and 3V (b). By increasing the voltage range from 1V to 2V or 3V, the current in reverse direction significantly increases while the forward current reaches saturation, decreasing the current rectification ratio. Since the reverse current increase appears at rather small voltages (at about 2V), the breakdown might be explained by Zener behavior rather than common avalanche breakdown, but further studies are required.

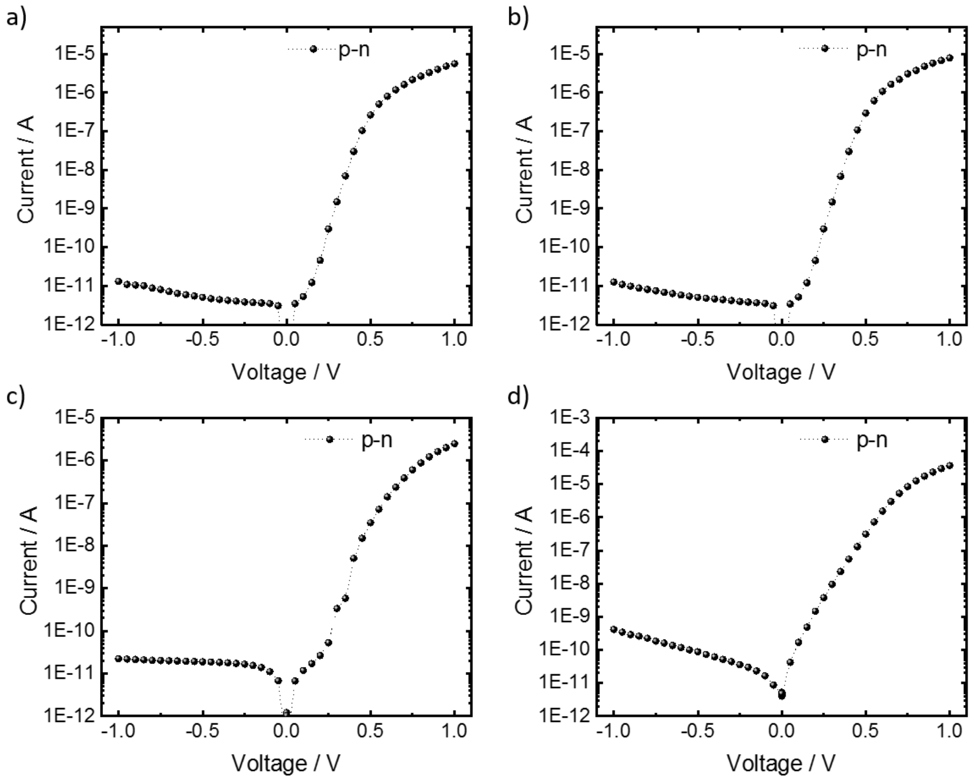


Figure S10: Current-voltage curves of the *p*-*n*-SURMOF of different samples. The current rectification varies between $0.9 \times 10^5$. and $6.4 \times 10^5$.

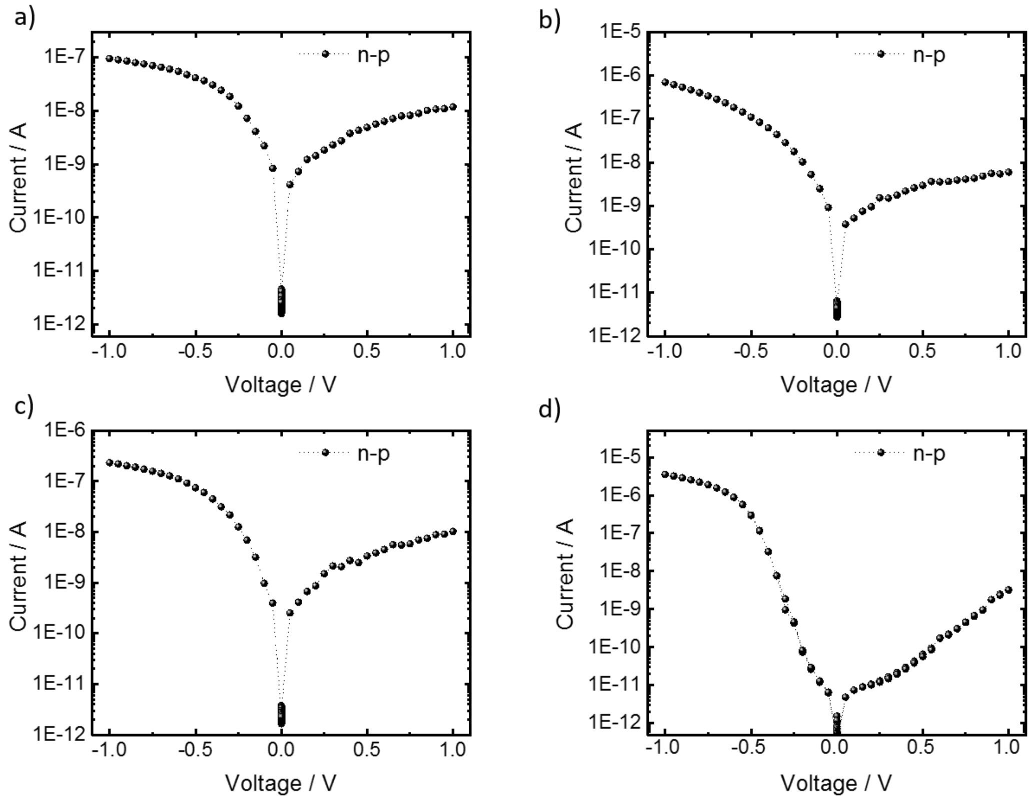


Figure S11: Current-voltage curves of the *n*-p-SURMOF of different samples. The current rectification varies between 8 and 1100.

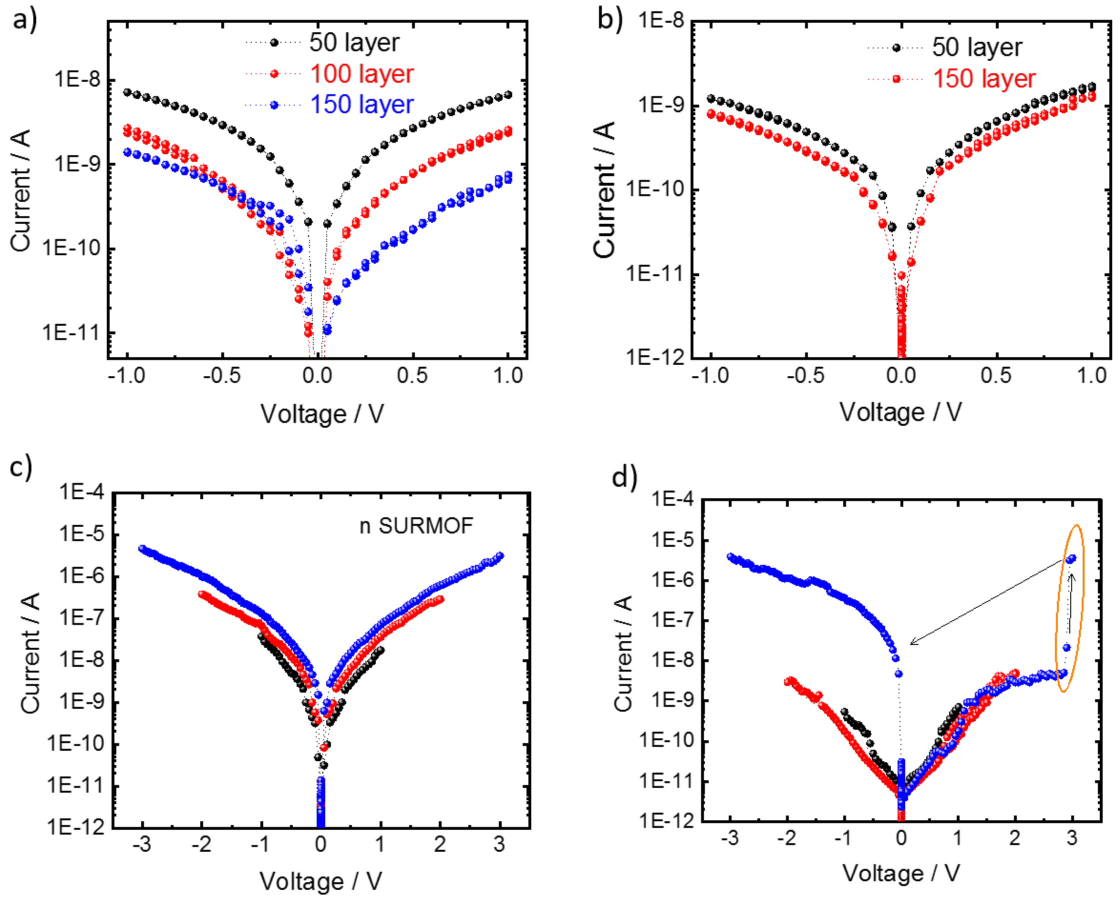


Figure S12: Current-voltage curve of pure *n*-SURMOFs (a) and *p*-SURMOFs (b) prepared with a different number of synthesis cycles, resulting in different thicknesses. The curves are measured in 2 consecutive voltage cycles.
Current-voltage curves of the *n*-SURMOF (c) and of the *p*-SURMOF (d) made with 50 synthesis cycles each, measured with voltage ranges from -1 to +1 V, -2 to +2 V and -3 to +3 V. In panel d), at about +2.9, the current suddenly increases by 3 orders of magnitude. The high current (i.e. high conductance) also remained when measuring the negative voltage branch. We assume the device broke down at the high voltage.
